# Knowledge of genetic test results among caregivers and individuals with spinal muscular atrophy

**DOI:** 10.1371/journal.pone.0276756

**Published:** 2022-11-08

**Authors:** Lisa Belter, Allison Mazzella, Shannon O’Brien, Jill Jarecki

**Affiliations:** 1 Research Department, Cure SMA, Elk Grove Village, IL, United States of America; 2 Division of Penn Translational Medicine and Human Genetics, Perleman Center for Advanced Medicine, Philadelphia, PA, United States of America; 3 Community Support Department, Cure SMA, Elk Grove Village, IL, United States of America; 4 Department of Strategic Risk Management, BioMarin Pharmaceutical Inc., San Rafael, CA, United States of America; Iowa State University, UNITED STATES

## Abstract

Spinal muscular atrophy (SMA) is a progressive recessive genetic disease. Early identification is critical for achieving maximal treatment benefit. Survival motor neuron (SMN) 2 copy number may be a needed descriptor of disease severity than SMA type. Therefore, we assessed knowledge of *SMN2* copy number among those with SMA and their caregivers via a phone survey. Only patients with SMA (or their caregivers) registered in the Cure SMA database with no *SMN2* copy number on file were eligible. Descriptive results are reported. Backward stepwise multinomial logistic regressions determined if specific factors predicted knowledge of *SMN2* copy number. Engagement with the SMA community (odds ratio [OR] 1.82; p<0.0001), ability to walk (OR 1.74; p = 0.006), and current age at time of survey (OR = 0.98; p<0.0001) each positively predicted knowledge of *SMN2* copy number. Of 806 completed surveys, the majority (n = 452; 56.3%) did not know *SMN2* copy numbers for themselves (n = 190; 62.5%) or their loved ones (n = 261; 52.4%). Of these, 66 respondents (8.2%) said genetic testing had not been done. Motor function increased linearly with increasing *SMN2* copy number. *SMN2* copy number is emerging as a critical descriptor of severity for SMA as type becomes more obsolete with early drug treatment. Communication of *SMN2* copy numbers is recommended as a standard part of the treatment plan.

## Introduction

Spinal muscular atrophy (SMA), a recessive genetic disease, causes progressive muscular atrophy, respiratory failure, and weakness, leading to death in severe cases [1]. It is caused by loss or mutation of the survival motor neuron (SMN) 1 gene [2]. A near duplicate gene, *SMN2*, encodes the same protein as *SMN1*, but it is a nonfunctional variant and the translated protein is mostly shortened and unstable [3–7]. Although *SMN2* is not a replacement for *SMN1*, research has demonstrated that it modifies the severity of the disease [5]. More copies of the *SMN2* gene increase the amount of full-length protein produced, which acts as a partial substitute for *SMN1*, modifying SMA severity [3]. In general, the higher the *SMN2* copy number, the less severe the symptoms of SMA [8].

Therapeutic research is focused on compounds that boost SMN gene expression [3]. In December 2016, the United States (US) Food and Drug Administration (FDA) approved the first SMA-specific therapy, nusinersen. Nusinersen is an antisense oligonucleotide *SMN2* pre-messenger RNA splicing modifier designed to increase production of the *SMN2* protein [9, 10]. In May 2019, the FDA approved the second SMA-specific therapy, onasemnogene abeparvovec-xioi, a gene therapy that replaces the missing or non-functioning *SMN1* gene [11, 12]. And most recently, a third SMA-specific therapy, risdiplam, an *SMN2* splicing modifier, was approved in August 2020 [13]. These therapies are changing the course of SMA. Compared with later initiation, early treatment with any of the approved therapies has been shown to lead to greater benefits in the form of increased motor function beyond expectations for a particular SMA type [3, 12, 14, 15].

To achieve maximal therapeutic benefit, early identification and treatment of affected infants in the pre-symptomatic period are critical [16]. Therefore, the need for reliable and well-validated newborn screening assays is paramount. In July 2018, the US federal government added SMA to the Recommended Uniform Screening Panel—the list of suggested conditions for which states should screen within their statewide universal newborn screening programs. Since then, approximately 90% of all states in the US have screened for SMA [17].

As effective treatments enable children with severe types of SMA to achieve motor milestones [3], the traditional designations of severity are becoming obsolete (**Table 1**). Rather than basing prognosis on the age of symptom onset and motor milestones achieved, *SMN2* copy number may offer an earlier and more accurate prediction [8]. For healthcare providers, earlier diagnosis enables earlier treatment initiation; for patients and their caregivers, knowing the *SMN2* copy number allows them to be more knowledgeable about their condition and prognosis and helps them become partners with their healthcare providers. Cure SMA maintains a voluntary database of patients with SMA. While *SMN2* copy numbers are among the details listed in the database, they are not available for all enrolled patients. To determine knowledge levels among patients with SMA and their caregivers regarding details of their condition, we surveyed members of the Cure SMA database who had no *SMN2* copy numbers recorded to collect self-reported information on *SMN2* copy numbers, including reasons members did not know them, SMA type, and current level of motor function.

**Table 1:** Traditional SMA classification [1].

Type	Approximate Proportion of Patients (%)	Age of Onset	Function
I	60	Within first 6 months	Children unable to sit, typically experience respiratory failure before 2 years of age
II	30	6–18 months	Children may sit on their own but unable to walk without assistance
III	15	3 years	Children can walk unassisted but may lose the ability and require a wheelchair
IV	<5	Teens or early 20s	Mild muscle weakness that progresses slowly, with adults usually requiring walking aids after age 60

## Patients and methods

### Cure SMA database

Cure SMA is the largest SMA patient advocacy organization based in the US, and it maintains the largest SMA database globally. The database contains demographics, SMA type, and patient experiences from a broad spectrum of patients [18]. All data in the database is caregiver or self-reported and not verified with clinical records. At the time of the survey, the database contained information from 8606 individuals with SMA. Most individuals (38.7%) had SMA type I, 30.8% had type II, 17.9% had type III, 3.0% had type IV, and the remaining 9.7% had an unknown or other SMA type with a different genetic cause (ie, non-5q SMA), such as Kennedy’s disease, SMA with respiratory distress, or distal SMA. *SMN2* copy number was missing from 87.7% of the database members. Among those who had an *SMN2* copy number reported, 42.4% reported 3 *SMN2* copies, 39.0% reported 2 copies, 11.7% reported 4 copies, 5.3% reported 1 copy, and 1.6% reported 5 or more copies (data on file). Enrollment in the database is voluntary [18] and by submitting personal information to Cure SMA, individuals acknowledge that Cure SMA may use the information to fulfil the mission of the organization. Therefore, data was not fully anonymized for the analysis and informed consent was not collected. De-identified data from the membership database can be provided from Cure SMA following a data request and committee review.

### Patients

In order to evaluate knowledge of *SMN2* copy number among the Cure SMA community, only patients with SMA (or their caregivers) who were registered in the Cure SMA database and had not previously reported their *SMN2* copy number to Cure SMA were eligible to participate in the telephone survey. All eligible survey participants lived in the US and had a listed phone number on file.

All respondents provided verbal informed consent at the start of the survey. Identifying information and responses were kept confidential and used only in the aggregate to generate disease statistics and generalized disease information. The survey qualified as exempt research by the Western Institutional Review Board and survey data was not made publicly available. Funding for the survey was provided by members of the 2020 Cure SMA Industry Collaboration.

### Survey

A 10-question phone survey was conducted by trained administrators reading from a prepared script. The administrators were employees of Snow Companies, a patient management company that supports direct-to-patient communications for research initiatives. All calls were placed between August 11, 2020, and October 29, 2020. As a thank you, each respondent received a $20 gift card.

Questions included the patient’s date of birth, diagnosis date of SMA, age when first SMA symptoms appeared, SMA genetic testing results, SMA type, *SMN2* copy number, and current motor function ability. SMA type was self-reported by respondents. Motor function was assessed through a series of yes/no questions regarding achievement of specific developmental milestones (**S1 File**).

### Analysis

Descriptive results are reported to describe the demographics of the survey responders and summarize the survey responses. Backward stepwise multinomial logistic regressions were used to determine if specific factors predicted whether or not someone would know their own (or child’s) *SMN2* copy number. The backward stepwise regression approach used a full model with all predictor variables included and removed predictor variables at each step that were the least significant in the model (**S2 File**). The explanatory variables that were used in the analysis were current age of the affected individual at time of survey, SMA type, year of SMA diagnosis, engagement with the Cure SMA community (defined as registering for a Cure SMA-sponsored event in the last 5 years), whether or not someone has been treated with a commercially available therapy at the time of the survey, geographic location (rural, urban, suburban, or town), current motor function (defined as a non-sitter, sitter, or walker), and relationship to affected individual (either self or caregiver/other). Both treatment information and engagement with the Cure SMA community were pulled from the Cure SMA membership database and merged with the survey data.

In addition to the structured questions, survey administrators wrote notes, compiling free-text comments from the respondents and the progress of each call. To determine if correlations existed between respondent demographics or other survey data, Cure SMA partnered with Thematics to perform the analysis [19]. Thematics is an online platform that uses artificial intelligence as well as human programming to extract group sentiments [19]. Thematics also performed statistical significance testing through 2-tailed tests to calculate volume differences. After the initial sentiment analysis was performed, Cure SMA modified the parameters with information specific to SMA and the survey design.

## Results

### Population

Of 2807 eligible households in the Cure SMA database contacted, 806 surveys were completed. The most common reason for nonresponse was failure to make contact (n = 1881), followed by refusal to participate (n = 120). Three completed surveys were excluded from the analysis because they had been completed on behalf of someone who did not have SMA. The final sample was composed of 803 respondents.

The majority of respondents were caregivers (62.1%) of someone affected with SMA. Nearly half (46.3%) of surveys were completed by or on behalf of someone with SMA type II (**Table 2**). A similar proportion of surveys were completed by or on behalf of someone with SMA type I (24.8%) or type III (20.7%), while the smallest proportion (1.4%) had SMA type IV. Half of the surveys (50%) were completed on behalf of an affected individual between 0 and 18 years of age.

**Table 2:** Demographics.

		SMA Type^a^ of the Affected Individual					
		Type I	Type II	Type III	Type IV	Other^b^	Unknown
Total, n (%)	803	199 (24.8)	372 (46.3)	166 (20.7)	11 (1.4)	30 (3.7)	25 (3.1)
Current age of affected individual, in years, mean (SD)	21.7 (16.7)	10.1 (10.9)	22.0 (14.5)	32.6 (17.1)	50.6 (17.1)	16.0 (10.3)	32.9 (19.1)
Relationship to affected individual, n (%)							
Caregiver^c^	499 (62.1)	179 (35.9)	229 (45.9)	56 (11.2)	0	22 (4.4)	13 (2.6)
Self	303 (37.7)	19 (6.3)	143 (47.2)	110 (36.3)	11 (3.6)	8 (2.6)	12 (4.0)
Other	1 (0.1)	1 (100)	0	0	0	0	0
Age at diagnosis, in years, mean (SD)	3.7 (8.8)	0.3 (2.4)	1.9 (4.1)	9.5 (12.3)	37.0 (21.5)	1.3 (2.0)	6.0 (11.7)
*SMN2* Copy Numbers, n (%)^d^							
1 copy	19 (5.4)	9 (7.9)	5 (3.5)	3 (4.0)	0	1 (14.3)	1 (20.0)
2 copies	146 (41.6)	88 (77.2)	35 (24.1)	17 (22.4)	1 (25.0)	4 (57.1)	1 (20.0)
3 copies	149 (42.5)	17 (14.9)	97 (66.9)	30 (39.5)	1 (25.0)	1 (14.3)	3 (60.0)
4 copies	30 (8.6)	0	6 (4.1)	22 (29.0)	2 (50.0)	0	0
5 or more copies	7 (2.0)	0	2 (1.4)	4 (5.3)	0	1 (14.3)	0

SD = standard deviation; SMA = spinal muscular atrophy

^a^Self-reported SMA Type

^b^Other SMA type refers to non-5q-linked SMA

^c^Caregivers include 490 parents, 4 relatives, 2 spouses, 2 grandparents, and 1 friend

^d^Among those who reported an *SMN2* copy number

Approximately 25% of respondents or their loved one were diagnosed without a genetic test, although 36% of these diagnoses occurred after genetic testing became available. In a stepwise logistic regression, for every increase in year, the odds of having a genetic test increased by 10.8%. On the other hand, having a less common type of SMA—such as type IV or a non-5q type of SMA—or not knowing SMA type decreased the odds of having a genetic test done by 79.8%, 66.1%, and 70.4%, respectively. A small proportion of respondents (5.1%) reported that they were diagnosed through a muscle biopsy, either alone or in conjunction with a genetic test. The most recent diagnostic biopsy was performed in 2018, and no follow-up genetic test was reported, therefore, *SMN2* copy number was unknown.

### SMN2 copy numbers

The majority of respondents (n = 452; 56.3%), noting that survey eligibility was based on missing information on *SMN2* copy number in the Cure SMA database, did not know *SMN2* copy numbers for themselves (n = 190; 62.5%) or their loved one (n = 261; 52.4%; **Table 3**). Of these, 66 respondents (8.2%) said genetic testing (unspecified) had not been done, and 48.1% cited other reasons, such as never being told from their healthcare provider, for not knowing their copy number. Among those who knew their *SMN2* copy number, the average age at diagnosis was 3.2 years (standard deviation [SD] 8.0 years), compared with 4.1 years (SD 9.5 years) for those who did not know their *SMN2* copy number. This difference in the average age at diagnosis was not statistically significant. Mean current age was younger for affected individuals with known *SMN2* copy number than those who did not know (18.7 years versus 24.1 years, respectively; p<0.0001).

**Table 3:** Demographics of affected individuals by knowledge of *SMN2* copy number.

	*SMN2* Copy Number		
	Known	Not Known	
		Genetic Testing Not Done	Other Reason^a^
		66 (8.2)	386 (48.1)
Total, n (%)	351 (43.7)	452 (56.3)	
Type of SMA, n (%)^b^			
Type I	114 (57.3)	85 (42.7)	
Type II	145 (39.0)	227 (61.0)	
Type III	76 (45.8)	90 (54.2)	
Type IV	4 (36.4)	7 (63.6)	
Other^c^	7 (22.6)	24 (77.4)	
Unknown	5 (20.8)	19 (79.2)	
Current age of affected individual, in years, mean (SD)	18.7 (16.9)	24.1 (16.2)	
Relationship to affected individual, n (%)			
Caregiver^d^	237 (47.6)	261 (52.4)	
Self	114 (37.5)	190 (62.5)	
Other	0	1 (100.0)	
Age at diagnosis, in years, mean (SD)	3.2 (8.0)	4.1 (9.5)	

SD = standard deviation; SMA = spinal muscular atrophy; *SMN2* = survival motor neuron 2 gene

^a^ “Other” reasons not collected in survey

^b^Percentages calculated by row

^c^Other SMA type refers to non-5q-linked SMA

^d^Caregivers include 490 parents, 4 relatives, 2 spouses, and 2 grandparents

Among respondents who did not know their *SMN2* copy number, 50.2% had SMA type II, 19.9% had type III, 18.8% had type I, and 1.6% had type IV. A small proportion (4.4%) reported they did not know their SMA type. Location (ie, rural, urban, suburban, or town) was similar between respondents who knew their *SMN2* copy number and those who did not, with the greatest proportion of respondents living in rural areas. SMA engagement, defined as participation in Cure SMA conferences and events, was similar across all *SMN2* copy numbers, ranging from 52% among those with 3 copies, 57% among those with 4 or 5 or more copies, to 58% among those with 1 or 2 copies. Among those who did not know their *SMN2* copy number, approximately a third was engaged, with 62% not engaged.

In general, *SMN2* copy numbers increased as disease severity decreased. The majority of respondents with type I SMA (77.2%) had 2 *SMN2* copies, while the majority of those with type II SMA (66.9%) had 3 *SMN2* copies. Among respondents with type III SMA, 22.4% had 2 *SMN2* copies, 39.5% had 3 copies, and 29.0% had 4 copies. Almost half of those with type IV SMA had 4 copies. Of those who knew their *SMN2* copy number but not their SMA type, 60.0% had 3 *SMN2* copies. Few respondents reported having 5 or more *SMN2* copies: only 1.4% of those with SMA type II and 5.3% of those with type III reported having 5 or more copies. No respondents with type I or IV reported 5 or more *SMN2* copies (**Table 2**).

### Motor function

**Fig 1:**
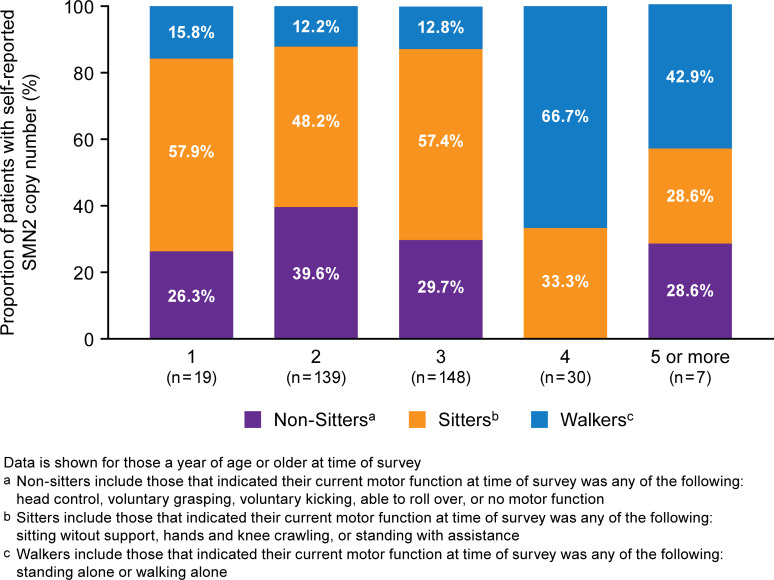
Motor function by self-reported *SMN2* copy number. Data is shown for those a year of age or older at time of survey. a Non-sitters include those that indicated their current motor function at time of survey was any of the following: head control, voluntary grasping, voluntary kicking, able to roll over, or no motor function. b Sitters include those that indicated their current motor function at time of survey was any of the following: sitting without support, hands and knee crawling, or standing with assistance. c Walkers include those that indicated their current motor function at time of survey was any of the following: standing alone or walking alone.

A large proportion of respondents reported motor functions atypical for their reported SMA type. For example, nearly a third of respondents (31%) with type I SMA were able to sit without support, and 5.1% of those with type II were able to walk unassisted at the time of the survey. Additionally, 4.6% with type I SMA reported the ability to walk alone. However, regardless of these atypical functions by SMA type, motor function generally increased with increasing *SMN2* copy number (**Fig 1**). While certain motor functions, such as walking, remained low for those with 1, 2, or 3 *SMN2* copies, the proportion of individuals achieving these functions rose markedly for *SMN2* copy numbers of 4 or 5.

### Factor analysis

We wanted to determine if several factors contributed to whether *SMN2* copy number was known or not known. Thus, a backward stepwise regression analysis was completed to assess which factors may predict whether a patient or caregiver knew the *SMN2* copy number (**Table 4**). The current age of the affected individual (odds ratio [OR] 0.98; p<0.0001), engagement with the Cure SMA community (OR 1.82; p<0.0001), and ability to walk (OR 1.74; p = 0.006) predicted whether or not an affected individual (or caregiver) knew their *SMN2* copy number. The year someone was diagnosed, whether or not they have been treated with an SMA-modifying therapy, the relationship the survey responder has with the affected individual, and the geographic location of the affected individual did not predict knowing the *SMN2* copy number of the affected individual. In a separate analysis, among those that knew their *SMN2* copy number, they began a SMA-modifying therapy younger (mean: 231.0 months; SD 226.5 months) than those who did not know their *SMN2* copy number (mean: 332.2 months; SD 206.2 months) (p = 0.012).

**Table 4:** Factors predicting knowing *SMN2* copy number.

Independent Variable	OR	95% CI	*p*-value
Current age, years	0.98	0.97–0.99	<0.0001
Engagement in SMA community			
No	1		
Yes	1.82	1.36–2.44	<0.0001
Motor function^a^			
Non-sitter	1		
Sitter	1.24	0.90–1.73	0.19
Walker	1.74	1.17–2.58	0.006

CI = confidence interval; OR = odds ratio; SMA = spinal muscular atrophy; *SMN2* = survival motor neuron 2 gene

^a^Current motor function at time of survey was defined as non-sitters (those that could do any of the following motor functions: head control, voluntary grasping, voluntary kicking, able to roll over, or no motor function); sitters (those that could do any of the following motor functions: sitting without support, hands and knee crawling, or standing with assistance); and walkers (those that could do any of the following motor functions: standing alone, walking with assistance, or walking alone)

Stepwise regressions were conducted to test potential predictor variables. Statistical significance was tested after each iteration and data from final best fit models are presented here. The following independent variables were excluded from the final method: year of diagnosis, treatment, relationship, and geographic location.

### Open-ended supplement

Survey administrators collected free-text notes on more than 2700 respondents and the progress of each call, regardless of whether or not the survey was completed. Cure SMA performed a sentiment analysis using the program Thematics on this information to identify any correlations with demographics or other collected survey information.

Of the households with which the survey administrators connected, 120 individuals declined to participate. Sentiment analysis elaborated on the reasoning behind this decision, with one-third of these individuals declining to participate in the survey because it did not apply to them and their family.

Of the survey respondents, sentiment analysis also explored diagnostic methods. A total of 137 participants had diagnostic methods in their free-text notes, and more specifically, 58 respondents (42.3%) identified muscle biopsy as their primary diagnostic method. The majority of this subpopulation (62.0%) identified muscle biopsy as their only diagnostic method, while others indicated muscle biopsy as their primary diagnostic method with another confirmatory method. Additionally, 30 respondents (49.2%) detailed that although genetic testing was not their primary diagnostic method, they had recently received a genetic test to determine treatment eligibility. The majority (73%) of these individuals were originally diagnosed prior to a genetic test becoming available (prior to 1995), but they did not discuss their original diagnostic method (**Table 5**).

**Table 5:** Open-ended supplement: Diagnostic methods information^a^.

	n (%)
Total^b^	137
Genetic test	61 (44.5)
Diagnostic genetic test	31 (50.8)
Genetic test at a later date for treatment^c^	30 (49.2)
Muscle biopsy^d^	58 (42.3)
Muscle biopsy only	36 (62.0)
Muscle biopsy and genetic test	17 (29.3)
Muscle biopsy and blood test	3 (15.5)
Muscle biopsy and EMG	1 (1.7)
Muscle biopsy and nerve conduction study	1 (1.7)
Bloodwork^e^	8 (6.0)
Bloodwork only	5 (62.5)
Bloodwork and genetic test	3 (37.5)
Other method	10 (7.6)
ECG^e^	1 (10.0)
ENG^e^	1 (10.0)
Nerve conduction study^f^	1 (10.0)
Other tests that are not genetic tests^f^	7 (70.0)

ECG = electrocardiogram; EMG = electromyogram; ENG = electroneurogram; SMA = spinal muscular atrophy

^a^Responses recorded verbatim

^b^Only respondents who had diagnostic methods stated in the free-text notes of their phone survey

^c^Patients diagnosed through a means other than genetic test, but then received a genetic test at a later date to be eligible for SMA therapy

^d^Among respondents who identified muscle biopsy as their primary diagnostic method

^e^Among respondents who identified bloodwork as their primary diagnostic method

^f^Among respondents who identified other methods as their primary diagnostic method

Of the respondents who had SMA diagnosed through muscle biopsy, nearly half had type II SMA (49.1%) and did not know their *SMN2* copy number (69.0%). Of note, 10 of these individuals were under 20 years of age, 4 of whom were 10 years or younger, and therefore, had muscle biopsy as their diagnostic method after genetic testing became available (**Table 6**). In reviewing these individuals, 5 received muscle biopsy along with genetic testing. Two individuals reported having a “rare form” of SMA leading to the muscle biopsy, while 1 child was originally misdiagnosed and later correctly diagnosed through muscle biopsy. The other 2 individuals stated they only received a muscle biopsy.

**Table 6:** Open-ended supplement: Demographics of respondents diagnosed through muscle biopsy.

	n (%)
*SMN2* copy number	55 (100)
1	0 (0.0)
2	6 (10.9)
3	9 (16.4)
≥4	2 (3.6)
Unknown	38 (69.0)
SMA type	55 (100)
Type I	5 (9.1)
Type II	27 (49.1)
Type III	14 (25.5)
Type IV	2 (3.6)
Other	4 (7.3)
Unknown	3 (5.5)
Current age, yrs	55 (100)
0–10	4 (7.3)
11–20	6 (10.9)
21–30	14 (25.5)
31–40	14 (25.5)
≥41	17 (30.1)
Age of diagnosis, yrs	55 (100)
0–10	47 (85.5)
11–20	4 (7.3)
21–30	2 (3.6)
31–40	2 (3.6)
≥41	0 (0.0)

SMA = spinal muscular atrophy; *SMN2* = survival motor neuron 2 gene

## Discussion

In this population of people with SMA who did not have *SMN2* copy numbers recorded in the Cure SMA database, the majority of those surveyed did not know their *SMN2* copy numbers, even though only a small percentage of these respondents cited lack of genetic testing as the reason. Even if genetic testing had been performed, it may have been done when testing was only for *SMN1* deletions and *SMN2* copy numbers were not counted or reported. While the response rate to the survey was 28.7%, it was substantially higher than the average 10% response rate for telephone surveys [20]. Additionally, the survey sample was taken from a limited population and was representative.

Results of the phone survey demonstrated *SMN2* copy numbers correlated with improved motor function and thus less severe disease, which is consistent with the literature [2, 21–23]. It is notable that a proportion of patients achieved motor function milestones beyond that expected for a particular SMA type, such as a third of patients with type I SMA being able to sit without support. However, reported SMA type from the caregiver or patient was not verified through clinical records so conclusions behind the large portion of atypical motor function achievement by SMA type could not be drawn. Achievement of motor function milestones atypical for SMA type may have been the result of treatment with an SMA disease-modifying therapy and the age of first treatment; however, concordance was not analyzed in this study.

A previous study by Calucho and colleagues, described the distribution of *SMN2* copy numbers by SMA type. Their cohort of unrelated Spanish SMA patients described that 86% of patients with SMA type I had 2 *SMN2* copies compared to 77% in the telephone survey. Additionally, 87% of patients with SMA type II and 64% of those with type III had 3 *SMN2* copies, compared with 66.9% and 39.5%, respectively, in the present study. These findings reinforce the importance of *SMN2* copy number as a predictor of disease severity, but *SMN2* is not the sole phenotypic modifier. For example, the variant *SMN* c.859G>C results in less severe SMA phenotypes and could explain the variance between *SMN2* copies and SMA type [4]. Additionally, *SMN2* copies can vary structurally between patients, indicating genetic variability within SMA [24]. Caregiver and patient knowledge, however, of additional modifiers of SMA phenotype was not assessed in this study.

Additionally, as previous clinical trials have shown that the SMA disease trajectory varies both by treatment and *SMN2* copy number [3, 12, 14, 15], it is important for family members to be aware of the *SMN2* copy number of their loved one to manage the family’s expectations of the SMA disease course. Characterizing which populations would be more likely to know their copy number helps identify which populations could be targeted for future educational opportunities. The results of the factor analysis indicated that those who were engaged in the SMA community, specifically those registering for Cure SMA-sponsored events within the last 5 years, were 82% more likely to know their *SMN2* copy number than those who were enrolled in the database but did not participate in Cure SMA events. Interestingly, previously Cure SMA-sponsored online surveys also resulted in about 50% of the survey responders not knowing their *SMN2* copy number, suggesting that this is a more widespread concern [25]. This highlights that regardless of survey approach, there is a need to engage more individuals in SMA-sponsored events for education and community.

A small proportion of the respondents who did not know their copy number (8%) were diagnosed without a genetic test despite the availability of such a test. Additionally, in the free-text analysis, it was noted that 4 affected individuals within the last 10 years received a muscle biopsy as a diagnostic method for SMA. While data were not collected on the reason a muscle biopsy was performed, these results do suggest that further clinician education is warranted. A previous study evaluating the knowledge among physicians in identifying the early signs of SMA found that only 52.7% of the clinicians in the study correctly indicated the need for genetic testing to make a definitive diagnosis of SMA [26]. Given the growing importance of *SMN2* copy number as a prognostic indicator, it is both encouraging that a significant proportion of patients with SMA do receive genetic testing as well as concerning that some patients fall through the diagnostic cracks. Additionally, while the specific SMA disease-modifying therapies for each participant was not disclosed in this study, it is important to note that a genetic test is required to receive any therapy, and therefore, all patients should be informed of their copy number results. A treatment plan should include increasing awareness among healthcare providers and caregivers of the importance of knowing a patient’s *SMN2* copy number.

The results of this survey should be viewed in light of several limitations. Enrollment in the Cure SMA database is voluntary, and those enrolled may be more engaged in their treatment than those not enrolled. However, it should be noted that Cure SMA engagement was similar across all *SMN2* copy number categories among respondents to the telephone survey. These results may not be generalizable to people with SMA not registered in a database or to those living outside the United States. It is important to note that SMA type, *SMN2* copy numbers, and motor function were self-reported by respondents. Responses may have been subject to inaccurate recall, or respondents may not have remembered being told their *SMN2* copy number. The responses were not verified against medical records and may have contained inaccuracies. Additionally, SMA type is now complicated by use and age at SMA drug treatment. SMA type may no longer align with earlier treatment particularly if responders are defining SMA type by their copy number instead of their highest achieved milestone as traditionally defined.

## Conclusions

As treatments become more effective and children reach previously unattainable motor milestones, the traditional SMA types are becoming obsolete and *SMN2* copy number is emerging as an earlier, more critical prognostic indicator. Earlier diagnosis allows for earlier treatment, which has been associated with greater benefit. Knowing a patient’s copy number also allows for more informed prognosis and enables patients and their caregivers to be active partners in SMA treatment. However, the results of this survey demonstrate that most individuals with SMA are unaware of their *SMN2* copy number, and in some cases, their type of SMA. Early awareness of *SMN2* copy number is critical to inform SMA therapy decisions and optimize outcomes, and determination of *SMN2* copy number should become a standard part of diagnosis.

## Supporting information

S1 File Cure SMA phone survey. (PDF)

S2 File Stepwise regression model. (XLSX)
