# Supplementary material for: Knowledge of genetic test results among caregivers and individuals with spinal muscular atrophy
Source: PLoS One. 2022 Nov 8;17(11):e0276756. doi: 10.1371/journal.pone.0276756 (PMC9642888; doi:10.1371/journal.pone.0276756)
Supplement: S1 File — (PDF) [file pone.0276756.s001.pdf]

Cure SMA SMN2 & Motor Function Survey

A. Greeting:

1. "Hello, my name is *[First name],* I'm calling from Snow companies, a patient engagement company, on behalf of Cure SMA.   Am I speaking with *[patient or caregiver name]*?
   - *(If "Yes", move to Section B, Ethics and Informed Consent)*
   - *(If "No,")* "Is *[patient or caregiver name]* available?"
     i. *(If "Yes",)* "May I speak to them now, please?"
        1. *(If "Yes", move to Section B, Ethics and Informed Consent)*
        2. *(If "No/Don't Know",)* "Is there a better time to call back to speak to them?"
           a. *(If "Yes",)* "Thank you. We'll call back then." [End Call]
           b. *(If "No" or "Don't Know",)* "Okay, I'll call back at another time. Have a wonderful day." [End Call]
   - *(If no adults in household,)* "I'm sorry, but we can only complete the survey with an adult with SMA or the primary caregiver of a person with SMA. Thank you for your time." [End Call]
   - *(If respondent refuses to answer,)* "Okay, thank you for your time. Have a wonderful day." [End Call]
   - *(If voicemail is reached,)* "Hello, *[patient or caregiver name],* I'm sorry I missed you today. I am calling on behalf of Cure SMA to see if you'd be interested in participating in a brief survey. As a thank you for your participation, we will send you a $20 Amazon gift card. You can reach me at *[phone #]* or I will try back at another time. Thank you and have a wonderful day." [End Call]

B. Ethics and Informed Consent:

1. "Hello, *[patient or caregiver name],* thank you for your time today! We're conducting a quick survey on behalf of Cure SMA with the goal of learning how SMA is changing with new treatments and standards of care. The survey should take about 5 minutes to complete. As a thank you for your time, for those that complete the survey we will email a $20 Amazon gift card.   Do you have a few minutes to do our survey?
   - *(If no, go to number 2)*
   - *(If yes, go to number 3)*

2. "When would be a better time to call back to conduct the interview?" [Set Callback Time and End Call. If Patient/Caregiver Does Not Want to Participate, Thank Them for Their Time and End Call.]

3. "Great! Let me take a couple of minutes to explain what you can expect in this Cure SMA survey. The survey is 10 questions long, and your answers will help clinicians, researchers, and our biotech and pharmaceutical partners better understand the experiences of patients and caregivers.

I'd like to tell you a little bit about how the survey information may be used. Your answers will be used anonymously, which means that we will not disclose your personal information or identity when we use your answers. Your answers will be combined with the answers of the other survey participants and summarized in a report that will be used to advance research, education, and further the goals of Cure SMA. This report may be shared with grant recipients, content providers, sponsors, service providers, and other third parties who work with Cure SMA.

Your personal information and responses will be kept confidential in accordance with our privacy policy. We may use your information in the aggregate to provide disease statistics and generalized disease information. Cure SMA also may use your information to contact you regarding clinical studies and trials and other patient engagement opportunities. Unless we have your consent, Cure SMA will not share your Personal Information with any person or entity other than those affiliated with Cure SMA, entities acting on behalf of Cure SMA and relevant third parties such as Cure SMA research grant recipients or as may be required by law."

4. Would you like to continue with the survey?
   - *(If "Yes",)* "I will begin the survey now." *(Move to section C)*
   - *(If "No",)* "Okay, thank you for your time." [End Call]

C. <u>Survey:</u>

1. Have you been diagnosed with SMA? Or are you caring for a child with SMA? If you're a caregiver, what is your relationship to the person living with SMA?
   ☐ Self
   ☐ Caregiver; Parent
   ☐ Caregiver; Grandparent
   ☐ Caregiver; Relative
   ☐ Caregiver; Spouse
   ☐ Caregiver; Friend
   ☐ Other

2. Please spell the first and last name of the person you're caring for with SMA or your first and last name if you have SMA. *(Record answer)*
   - *(If a parent has more than one child with SMA)* I'm going to run through these questions for each of your children; we'll answer the questions for one child, and then restart the survey for your other child(ren). *(Record answer)*

3. What is *(your/your child's/your loved one's)* date of birth? *MM/DD/YYYY*

4. What is *(your/your child's/your loved one's)* date of diagnosis? *MM/DD/YYYY*

5. At what age, in months, did you notice that (you/your child/your loved one) was having SMA symptoms?

   ____

   ☐ Don't know

6. Were *(you/your child)* diagnosed with SMA by a genetic test?
   ☐ Yes
   ☐ No
   ☐ Don't know

7. When *(you/your child/your loved one)* were/was diagnosed, what type of SMA did a healthcare provider say *(you/your child/your loved one)* had?
   ☐ Type 0
   ☐ Type I
   ☐ Type II
   ☐ Type III
   ☐ Type IV
   ☐ I don't know
   ☐ Other

8. How many SMN2 copies do *(you/your child/your loved one)* have? SMN2 is a gene that produces the SMN protein, and people can have multiple copies of this gene.
   ☐ 1
   ☐ 2
   ☐ 3
   ☐ 4
   ☐ 5 or more
   ☐ I don't know
   ☐ I don't know because there was no genetic testing done.

9. Next, I'm going to read a list of motor functions. After each motor function, please tell me with either a "yes" or "no" answer if *(you/your child/your loved one)* can <u>currently</u> perform that function.
   - Head Control, in other words, being able to hold their head up on own
     ☐ Yes
     ☐ No
     ☐ Don't know
   - Voluntary grasping, in other words, being able to pick things up using their thumbs voluntarily versus grasping by reflex.
     ☐ Yes
     ☐ No
     ☐ Don't know

- Voluntary kicking
  ☐ Yes
  ☐ No
  ☐ Don’t know
- Roll over completely
  ☐ Yes
  ☐ No
  ☐ Don’t know
- Sitting without support
  ☐ Yes
  ☐ No
  ☐ Don’t know
- Hands and knees crawling
  ☐ Yes
  ☐ No
  ☐ Don’t know
- Standing with assistance, in other words, using a device to help with standing
  ☐ Yes
  ☐ No
  ☐ Don’t know
- Walking with assistance, in other words, using a device to help with walking
  ☐ Yes
  ☐ No
  ☐ Don’t know
- Standing alone
  ☐ Yes
  ☐ No
  ☐ Don’t know
- Walking alone
  ☐ Yes
  ☐ No
  ☐ Don’t know

10. Do you receive email updates from Cure SMA?
    a. *(If “Yes,” move to section D)*
    b. *(If “No”,)* “Would you like to be on their email list?”
        i. *(If “Yes,”)* “What is your preferred email address?” (Record answer and move to section D)
        ii. *(If “No,” move to section D)*

D. <u>Closing:</u>

“That concludes this survey. Thank you again for your time. Your responses will go a long way in helping us understand the impact of SMA. Also, as a thank you for your time, may I have your email address so we may email you a $20 gift card? *(Record answer)*

If you have any questions or concerns after this call, please do not hesitate to reach out to Cure SMA at research@curesma.org. Thank you again for your time and have a wonderful day!”
